# Carbon Dioxide Solubility in Three Bis Tri (Fluromethylsulfonyl) Imide-Based Ionic Liquids

**DOI:** 10.3390/molecules29122784

**Published:** 2024-06-11

**Authors:** Eric Quaye, Amr Henni, Ezeddin Shirif

**Affiliations:** 1 Industrial Systems Engineering, Faculty of Engineering and Applied Science, University of Regina, Regina, SK S4S 0A2, Canada; enq014@uregina.ca; 2 Process Systems Engineering, Faculty of Engineering and Applied Science, University of Regina, Regina, SK S4S 0A2, Canada; 3 Energy Systems Engineering, Faculty of Engineering and Applied Science, University of Regina, Regina, SK S4S 0A2, Canada; ezeddin.shirif@uregina.ca

**Keywords:** ionic liquids, intelligent gravimetric analyzer (IGA), CO_2_ capture

## Abstract

This study delves into the necessity of mitigating carbon dioxide (CO_2_) emissions, focusing on effective capture methods to combat global warming by investigating the solubility of CO_2_ in three ionic liquids (ILs), 1-Decyl-3-MethylimidazoliumBis (Trifluromethylsulfonyl Imide) [IL1], 1-Hexadecyl-3-Methyl imidazoliumBis (Trifluromethylsulfonyl Imide) [IL2] and Triethytetradecyl Ammonium Bis (Trifluromethylsulfonyl Imide) [IL3]. Solubility experiments were conducted at (30, 50 and 70) °C with pressures up to 1.5 MPa. The research shows [IL2] as the superior candidate for CO_2_ capture, with its longer alkyl chain, and is confirmed by its lower Henry’s Law constant. Utilizing the Peng Robinson equation of state, the study correlates well with the solubility measurements using three mixing rules. The study reveals promising results for IL1, IL2 and IL3 surpassing all other published ionic liquids including Selexol/Genesorb 1753, except for 1-Methyl-3-octylimidazolium bis(trifluoromethylsulfonyl)imide. Insights into the enthalpy and entropy of absorption underscore the significant impact of IL structure on CO_2_ solubility, emphasizing the potential of tailored ILs for advanced carbon capture strategies. In summary, this research highlights [IL2] as the optimal choice for CO_2_ capture, offering valuable contributions to the ongoing efforts in combating climate change.

## 1. Introduction

In the past two decades, the escalation of temperatures, largely because of the persistent elevation of atmospheric CO_2_ levels, has presented noteworthy concerns for the world in general [1]. This phenomenon has prompted widespread apprehension among researchers, scientists, and environmentalists regarding the broader implications of climate change. Consequently, there is a concerted effort within the research community to develop improved solvents or technologies capable of efficiently capturing CO_2_. A significant amount of CO_2_ release originates directly from the burning of fossil fuels, particularly hydrocarbons like natural gas, oil and coal. Various technologies, including membrane separation/permeation, adsorption, absorption, and cryogenic distillation, are utilized for carbon dioxide (CO_2_) capture from natural gas or flue gases. Among these methods, absorption, noted for its cost-effectiveness and energy efficiency, is widely adopted on a large scale [2].

Over the years, numerous mechanisms and processes have been explored for CO_2_ absorption, contingent upon the partial vapor pressures of the gas mixture. According to Zhang (2021) [2], physical solvents and amines have garnered significant attention due to their effectiveness and relatively lower costs. Physical solvents, in particular, offer advantages over amines, notably requiring less energy for solvent regeneration and exhibiting non-corrosive properties that prolong equipment lifespan, thus reducing maintenance expenses. Ionic liquids can be used for natural gas sweetening and mixed with amines or used in flue gas treatment.

Ionic liquids (ILs) represent a category of physical solvents that utilize intermolecular forces or functional group incorporation for CO_2_ capture. Their intrinsic properties, including low volatility, high thermal stability, and chemical resilience, offer distinct advantages. Minimal volatility facilitates nearly solvent-loss-free regeneration processes, thereby reducing operating costs. Additionally, high thermal and chemical stability minimizes degradation and corrosion risks, ensuring prolonged equipment durability. This technology addresses shortcomings observed in chemical solvents like Monoethanolamine (MEA), such as excessive energy consumption and solvent loss [3], potentially serving as a potential MEA substitute [4].

Ionic liquids are typically classified into two types: task-specific ILs (TSILs) and non-functionalized room temperature ILs (RTILs). The key difference lies in RTILs’ ability to function as conventional physical absorbents, governed by Henry’s Gas constant, whereas TSILs demonstrate both chemical and physical CO_2_ solubility. This dual capability enhances TSILs’ capacity for CO_2_ capture, as elucidated by Vadillo et al. (2022) [5].

The objective of this investigation is to assess the performance of three ionic liquids (ILs), categorized as promising physical solvents, in capturing CO_2_ across a range of temperatures (30 °C to 70 °C) and pressures relevant to industrial applications, up to 1.5 MPa utilizing an intelligent gravimetric microbalance (IGA-003).

## 2. Results and Discussion

### 2.1. CO_2_ Solubility Validation Test

**Figure 1:**
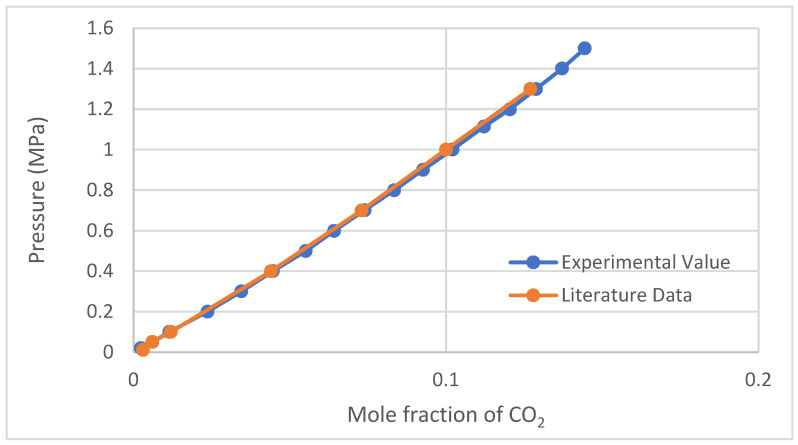
CO_2_ solubility validation with 0.0033% deviation from Shiflett and Yokozeki (2005) [6] at 323.15 K.

Similar to the density validation, tests were conducted to confirm the repeatability of the IGA-003 and its adherence to the relevant experimental Standard Operating Procedures (SOPs). During this validation phase, the IL, [BMIM][BF4], was employed to evaluate CO_2_ absorption. A comparative study was performed, aligning the obtained values with those documented by Shiflett and Yokozeki (2005) [6], who employed the same ionic liquid and referenced density values from a publication (Figure 1).

### 2.2. ILs CO_2_ Solubility

**Figure 2:**
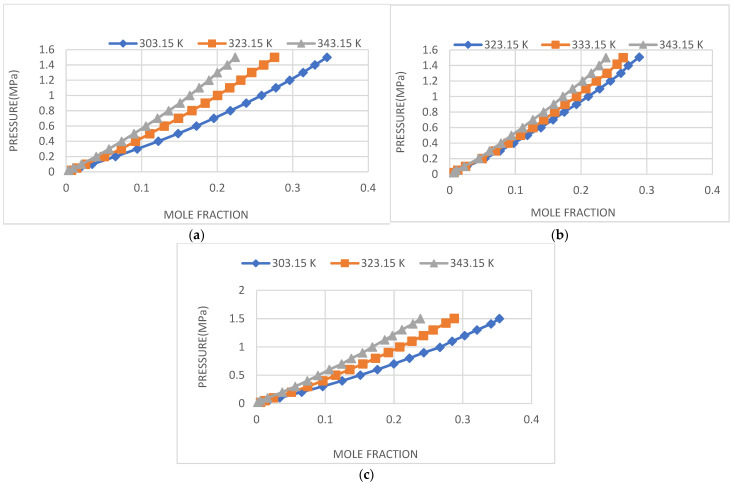
(**a**) CO_2_ solubility in IL1; (**b**) CO_2_ solubility in IL2; (**c**) CO_2_ solubility in IL3.

The CO_2_ solubility of IL1, IL2 and IL3 was measured at various temperatures and pressures up to 1.5 MPa. The obtained data for the two ILs were plotted in Figure 2 and recorded in Table 1 below.

### 2.3. Simulation Interaction Parameters

The experimental data were correlated using three mixing rules: (a) PR + vdW1, (b) PR + vdW2, and (c) PR + WS + NRTL. Table 2 below provides an overview of the calculated interaction binary parameters and their corresponding average absolute deviations (AADs %) for each mixing rule. The average absolute deviations (AADs %) for the vdW1, vdW2, and WS-NRTL rules applied to CO_2_ absorption in these ILs were determined to be 6.52%, 0.67%, and 0.62%, respectively, for IL1, 6.21%, 1.16%, and 1.10% for IL2, and 5.14%, 0.99%, and 0.94% for IL3. Notably, the Wong–Sandler (WS-NRTL) mixing rule exhibited the lowest average absolute deviation in both cases, indicating its superiority as the preferred choice among these options.

### 2.4. Henry’s Law Constant, Enthalpies and Entropies

The Henry’s Law constants (H) were evaluated through a comprehensive analysis involving the plotting of fugacity against mole fraction and the subsequent fitting of a second-order trend line. This analysis enabled the derivation of the slope of the second-order equation, which serves as an essential parameter in determining Henry’s Law constant [7]. Similarly, the estimation of entropy involved plotting the natural logarithm of Henry’s Law constant against the natural logarithm of temperature (T) for each temperature of the ionic liquid. The resulting negative enthalpy variation signifies an exothermic absorption process. The operation of the absorption of a gas in a liquid results in a decrease in the volume of the molecules from the gas phase to the liquid phase (equivalent to compression), leading to a reduction in the entropy. The negative entropy variation with the increase in temperature, as illustrated in Table 3 below, results from the decrease in the number of absorbed CO_2_ moles and a reduction in the solubility.

**Figure 3:**
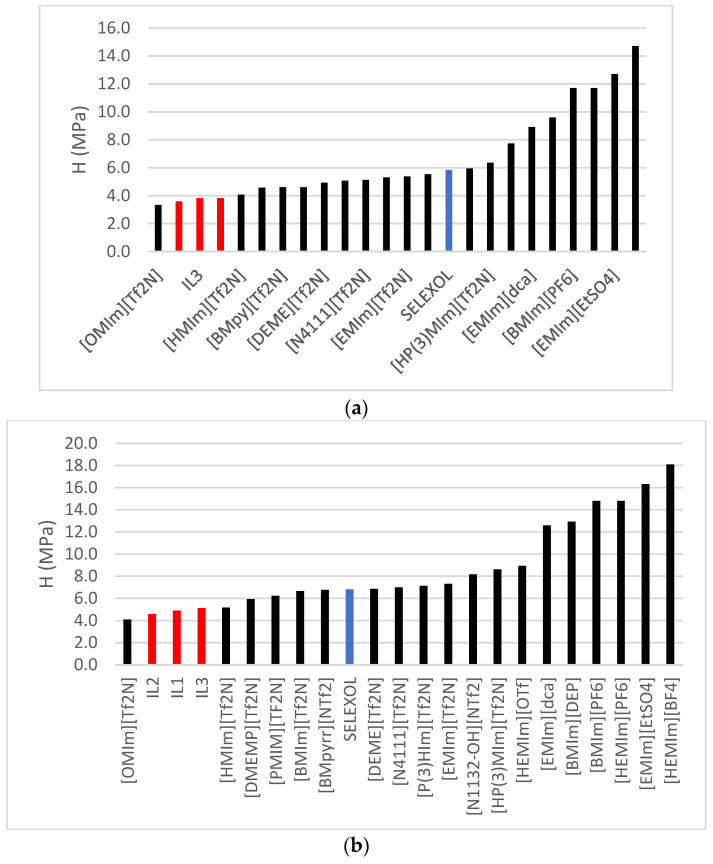
Henry’s Law constant comparison for CO_2_ solubility between ILs studied in this research against published ILs [7] and Selexol/Genesorb 1753 [8] at (**a**) 323.15 K and (**b**) 343.15 K.

A relatively lower Henry’s Law constant (H) implies the IL’s capability to absorb a larger quantity of CO_2_. In this study, [IL2] demonstrated the highest CO_2_ absorption capacity, while [IL1] exhibited the least. When juxtaposing the estimated Henry’s Law constants from this research with those of other ILs documented by previous researchers, [OMIM] [TF2N] was the only published IL with the highest solubility among those reported in this study, as seen in Figure 3 below.

**Figure 4:**
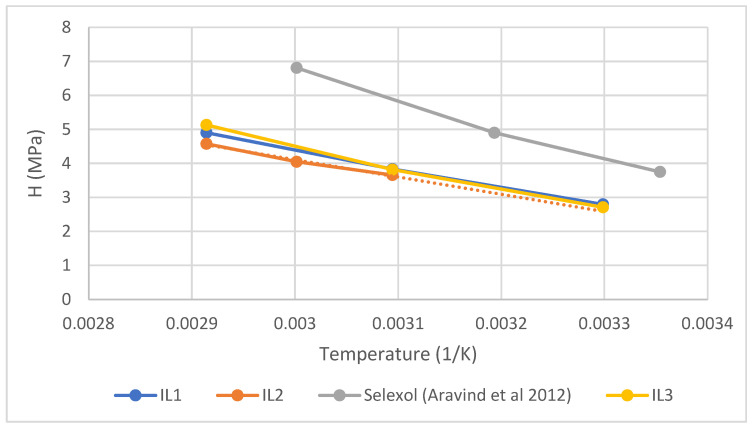
Comparison of Henry’s Law constants for CO_2_ in ILs obtained in this work and Selexol as reported by Aravind et al. (2012) [8].

Figure 4 showcases a notable CO_2_ absorption capacity exhibited by the studied ionic liquids in comparison to Selexol/Genesorb 1753 [8], a high-performance solvent utilized in gas treatment facilities. Given their superior CO_2_ absorption performance, the ILs investigated in this research can be regarded as promising solvents.

## 3. Materials and Method

### 3.1. Materials

Table 4 lists the detailed ILs used in this research along with their purity, chemical structures and nomenclature.

### 3.2. Density Measurement

**Figure 5:**
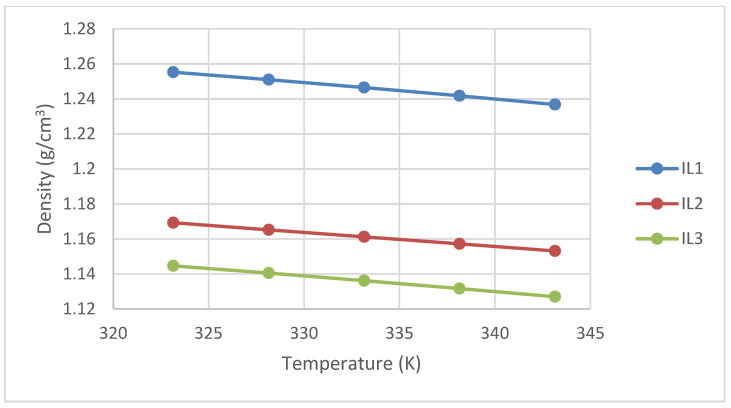
Densities of the ILs used in this work.

Before and after each experiment, at the corresponding temperature, the Anton Paar Density and Speed of Sound instrument (DSA 5000, Anton Paar, Graz, Austria) was tested with air and double-distilled water at atmospheric pressure to ensure the error was less than 0.00005 g·cm^−3^ when compared to the reference values for water and air stored in the instrument’s database. To further ensure the accuracy of the density measurements for the ILs utilized in this study, the density (*m*/*v*) of N-methyldiethanolamine (MDEA) with a concentration purity of ≥99% was initially determined using the Anton Paar density meter. These measured data were then compared with those published by Karunarathne et al. (2020) [7] for MDEA with a similar purity level. The comparison revealed an average deviation (AAD) of 0.014%, indicating excellent repeatability/accuracy in the density measurements. Subsequently, the densities of three specific ILs were determined at atmospheric pressure conditions (P = 98.3 kPa) and temperatures ranging from 20 °C to 70 °C using the density meter. The obtained data are provided in Figure 5 and Table 5 below.

### 3.3. Solubility Analysis

The IGA-003 analyzer, manufactured by Hiden Isochema Ltd. (Warrington, UK), incorporates cutting-edge components to facilitate gas sorption experiments effectively. A Polyscience water bath, providing precise temperature control within the reactor chamber, up to 343 K in this study, ensures optimal conditions for accurate gas sorption measurements. This system integrates a water jacket around the reactor chamber to provide equal heat/temperature all around. Within the core vessel of the reactor chamber, the IL is placed, creating a controlled environment for the experimental procedure. The experiment gas—carbon dioxide (CO_2_)—is introduced into the controlled system to study their sorption characteristics.

Pressure regulation maintains precise pressure conditions for gas injection into the reactor chamber. The Mass Flow Control (MFC) system plays an important role in controlling the rate of experiment gases getting into the reaction chamber, maintaining uniform gas flow for accurate absorption study. The IGA-003 microbalance takes note of the change in ionic liquid mass during gas absorption, providing essential data on ILs’ sorption behavior. In addition to the microbalance (IGA-003), a weight counterbalance enclosure compensates the weight of the reaction chamber and other components like the weight of the string, buoyancy, etc., allowing the IGA to measure specific weight changes in the sample material only during gas sorption precisely and accurately. The experimental procedure is automated and overseen by the data acquisition (DAQ) and control system, ideally operated through an onsite computer. This integrated system manages variables such as weight changes, pressure, gas flow, and temperature, maintaining the repeatability, efficiency, and accuracy of experiments performed with the IGA-003.

### 3.4. Thermodynamic Modelling

We utilized the Peng–Robinson Equation of State (PR EoS), as indicated in Equation (1), a well-established model renowned for its accuracy in describing the phase behavior of fluid mixtures, particularly at elevated pressures and temperatures. Through this model, we were able to estimate the absorption of CO_2_ over a range of pressures up to 1.5 MPa and temperatures reaching 70 °C. The selection of the PR EoS was deliberate, as it leverages critical components of both liquids and gases involved in the system, aligning closely with our research objectives.

(1) $$P=\frac{RT}{v-b_{m}}-\frac{a_{m}\left(T\right)}{v\left(v+b_{m}\right)+b_{m}\left(v-b_{m}\right)},$$

The coefficients of this model were predicted using various mixing rules, as stated below [7]:

- van der Waals one single binary interaction parameter;
- van der Waals two binary interaction parameters;
- NRTL model combined with Wong–Sandler mixing rules (WS-NRTL).

#### 3.4.1. van der Waals Mixing Rules

Two distinct mixing rules devised by van der Waals, namely van der Waals two (vdW2) and van der Waals one (vdW1), were employed to estimate the mixture variables represented by a_m_ and b_m_ [9]. The vdW1 mixing rule involves estimating a single interaction parameter (lij), whereas vdW2 entails estimating two (2) binary interaction parameters (lij and kij). The parameter a_m_ for vdW2 and vdW1 mixing rules was determined using Equation (2), while Equation (5) facilitated the estimation of a_ij_ based on the temperature-dependent binary interaction parameter, kij. The co-volume factor bm for both vdW2 and vdW1 was estimated through Equations (3) and (4), respectively. Furthermore, the interaction parameter bij was determined using Equation (6).

(2) $$a_{m}=\sum_{i}\sum_{j}x_{i}x_{j}a_{ij}$$

(3) $$b_{m}=\sum_{i}x_{i}b_{i}$$

(4) $$b_{m}=\sum_{i}\sum_{j}x_{i}x_{j}b_{ij}$$

where

(5) $$a_{ij}=\sqrt{a_{ii}a_{jj}}\left(1-k_{ij}\right)$$

(6) $$b_{ij}=\frac{b_{i}+b_{j}}{2}\left(1-l_{ij}\right)$$

NRTL model combined with Wong–Sandler mixing rule (WS-NRTL).

The modeling approach in this study integrates Wong–Sandler mixing rules, incorporating Equations (7) and (8) to determine the liquid–gas mixture’s attractive force parameter ‘a’ and co-volume parameter ‘b’. Utilizing the Non-Random Two Liquid (NRTL) model, the activity coefficient and excess Gibbs energy are computed, as specified in Equations (13)–(19). Binary interaction parameters (*τ*_ji, *τ*_ij, *τ*_ij_kij) are instrumental in estimating the mixture parameters, where *τ*_ji and *τ*_ij represent the NRTL parameters, and *g*_ij and *g*_jj denote the interaction energies between molecules ‘i’ and ‘j’, as elucidated by Nath and Henni (2020) [9]. Additionally, the value of α in Equations (14) and (15) is arbitrarily set at 0.3 in this study.

(7) $$a=RT\left(\frac{QD}{1-D}\right)$$

(8) $$b=\left(\frac{Q}{1-D}\right)$$

(9) $$Q=\sum_{i}\sum_{j}x_{i}x_{j}b_{ij}\left(b-\frac{a}{RT}\right)$$

(10) $$D=\sum_{i}x_{i}\left(\frac{a_{i}}{b_{i}RT}\right)+\left(\frac{G^{ex}}{CRT}\right)$$

(11) $$C=-lnln\left(\left(1+\sqrt{2}\right)/\sqrt{2}\right)$$

(12) $${\left(b-\frac{a}{RT}\right)}_{ij}=\frac{1}{2}\left[\left(b_{i}-\frac{a_{i}}{RT}\right)+\left(b_{j}-\frac{a_{j}}{RT}\right)\right]\left(1-k_{ij}\right)$$

(13) $$\frac{G^{ex}}{RT}=x_{i}x_{j}\left(\frac{\tau_{ji}G_{ji}}{x_{i}+x_{j}G_{ji}}+\frac{\tau_{ij}G_{ij}}{x_{j}+x_{i}G_{ij}}\right)$$

(14) $$G_{ij}=exp(-\alpha_{ij}\tau_{ij})$$

(15) $$G_{ji}=exp(-\alpha_{ij}\tau_{ji})$$

(16) $$\tau_{ij}=\frac{\left(g_{ij}-g_{jj}\right)}{RT}$$

(17) $$\tau_{ji}=\frac{\left(g_{ji}-g_{ii}\right)}{RT}$$

(18) $$ln\gamma_{i}=x_{j}^{2}\left[\tau_{ji}{\left(\frac{G_{ji}}{x_{i}+x_{j}G_{ji}}\right)}^{2}+\frac{\tau_{ij}G_{ij}}{{\left(x_{j}+x_{i}G_{ij}\right)}^{2}}\right]$$

(19) $$ln\gamma_{j}=x_{i}^{2}\left[\tau_{ij}{\left(\frac{G_{ij}}{x_{j}+x_{i}G_{ij}}\right)}^{2}+\frac{\tau_{ji}G_{ji}}{{\left(x_{i}+x_{j}G_{ji}\right)}^{2}}\right]$$

#### 3.4.2. Critical Properties Calculations

To utilize the model effectively, it is imperative to possess thermo-critical properties for both the gas and the solvent under investigation. In our study, we employed the modified Lydersen–Joback–Reid group contribution method [9] to ascertain the critical temperature (Tc), the acentric factor (ω) and the critical pressure (Pc) of the ILs. The Lydersen–Joback–Reid method is a chemical group contribution method revisited and updated by Valderrama and Rojas [10] for the prediction of the critical properties and the acentric factor of the ionic liquids. The full description and an Excel file are reported in reference [10]. The following equations are used:

(20) Tb (K)=198.2+ΣnΔTb

(21) $$\mathrm{Tc}\;(K)=\mathrm{Tb}/([A+B\Sigma n\Delta \mathrm{Tc}-\Sigma n\Delta {\mathrm{Tc}}^{2}])$$

(22) $$\mathrm{Pc}\;(\mathrm{bar})=M/({\left[C+\Sigma n\Delta Pc\right]}^{2})$$

(23) $$\omega =\frac{\left(T_{b}-43)(T_{c}-43\right)}{\left(T_{c}-T_{b})(0.7T_{c}-43\right)}\log\left[\frac{P_{c}}{P_{b}}\right]-\frac{\left(T_{c}-43\right)}{\left(T_{c}-T_{b}\right)}\log\left[\frac{P_{c}}{P_{b}}\right]+\log\left[\frac{P_{c}}{P_{b}}\right]-1$$

where A = 0.5703, B = 1.0121, C = 0.2573, D = 6.75 and Pb = 1.01325 bar.

In the equations, M is in g/mol, Tb and Tc are in K and Pc in bar, and n is the number of different groups. A table in reference [9] provides the values of Mi, ΔTc, ΔPc, ΔT_b_ for a variety of chemical groups for ILs with and without rings. Table 6 shows a summary of the critical properties of the ionic liquids studied.

### 3.5. Binary Interaction Parameter Optimization

The thermodynamic models employed in this research were developed by Dr. Nath and Dr. Kazi as reported in reference [9] using the MATLAB R2024a software, utilizing a bubble point algorithm. As illustrated in Equation (24), the optimization of binary interaction parameters was carried out using the Nelder–Mead simplex method, utilizing the ‘fminsearch’ function integrated within MATLAB to minimize the error in the objective function. Given the likelihood of experimental data inaccuracies at lower pressures, the optimization of binary interaction parameters was conducted within a pressure range of 0.1 to 1.5 MPa.

(24) $$Err=\frac{100}{n}\sum_{i=1}^{n}\left[\frac{P_{Exp,i}-P_{Cal,i}}{P_{Exp,i}}\right]$$

### 3.6. Henry’s Law Constant, Enthalpy of Absorption and Entropy of Solvation

Henry’s Law constant (H) for the two ILs was evaluated by analyzing the slope of the second-order polynomial of a plot with the mole fractions (x) against the fugacity (f) of CO_2_ at all temperatures. This constant, denoted as H_i_, represents the ratio of the solute (i) fugacity to its mole fraction in the solvent (j) at infinite dilution, occurring at a specific temperature within a specific pressure scope. Within this research, $f_{i}^{V}\;\mathrm{and}\;f_{i}^{L}$ signify the solute’s fugacity in the vapor and liquid phases, respectively, while $y_{i}$ and $x_{i}$ imply the mole fractions of the gas in the vapor and liquid phases, respectively, as outlined by Huseynov (2014) [11]. After the Henry’s Law constant (H) is determined from the absorption data points, the subsequent procedure involves evaluating the entropy of solvation (ΔS^∞^) at infinite dilution and the enthalpy of absorption (ΔH^∞^) at infinite dilution using the provided equations.

(25) $$H_{i}=\underset{x_{i}\to 0}{\lim}\frac{f_{i}^{L}\left(T,P,x_{i}\right)}{x_{i}}=\underset{x_{i}\to 0}{\lim}\frac{f_{i}^{V}\left(T,P,y_{i}\right)}{x_{i}}$$

(26) $$∆H^{\infty }=R{\left(\frac{\partial \left(\ln H\right)}{\partial \left(1/T\right)}\right)}_{P}$$

(27) $$∆S^{\infty }=-R{\left(\frac{\partial \left(\ln H\right)}{\partial \left(\ln T\right)}\right)}_{P}$$

## 4. Discussion and Conclusions

The observed trends in Henry’s Law constants for CO_2_ absorption in IL1, IL2 and IL3 reveal a consistent increase with temperature. This thermal dependence suggests the enhanced solubility of CO_2_ gas in ionic liquids at lower temperatures, aligning with typical gas solvation behavior.

The alkyl chain lengths and anion fluorination play crucial roles in determining the absorption behavior of CO_2_ in the studied ionic liquids (IL1, IL2 and IL3). IL1, composed of 1-decyl-3-methylimidazolium bis(trifluoromethylsulfonyl) imide, possesses a shorter alkyl chain compared to both IL2 and IL3. This difference in alkyl chain length significantly impacts the solvation properties of the ionic liquids, influencing their interaction with CO_2_ molecules.

The observed trends in Henry’s Law constants reveal interesting insights into the effect of alkyl chain length on gas solubility. Generally, relatively longer alkyl chains, as in IL2, tend to promote stronger interactions with gas molecules, resulting in lower Henry’s Law constants compared to shorter alkyl chain counterparts, such as IL1 and IL3. This phenomenon can be attributed to the increased surface area and van der Waals interactions provided by longer alkyl chains, facilitating greater solubility of CO_2_ in IL2.

Furthermore, the fluorination of the anion in IL1, IL2 and IL3 introduces additional effects on gas solvation behavior. The presence of fluorine atoms in the anion enhances the polarity and can induce specific interactions with gas molecules, contributing to the overall solvation process. This effect is evident in the observed Henry’s Law constants, where IL1, IL2 and IL3 exhibit impressive performance in terms of CO_2_ absorption compared to non-fluorinated counterparts reported in the published literature.

Regarding the obtained thermodynamic parameters, the negative enthalpy values (∆H^∞^) obtained for CO_2_ absorption in IL1, IL2 and IL3 indicate exothermic solvation processes. The enthalpy value calculated was −12.80 kJ/mol in IL1, −10.33 kJ/mol in IL2 and −13.79 kJ/mol in IL3, further confirming the exothermic nature of the absorption processes. Additionally, the negative entropy values imply a decrease in disorderliness within the system upon solvation, reflecting the ordered arrangement of solvent molecules around the solute during absorption.

In conclusion, the alkyl chain lengths and anion fluorination effect significantly influence the absorption behavior of CO_2_ in IL1, IL2 and IL3. The observed trends in Henry’s Law constants highlight the importance of molecular structure and interactions in gas solvation processes. The exothermic nature of the absorption processes and the decrease in entropy upon solvation further underscore the thermodynamic aspects of gas solvation in ionic liquids.

Comparative analysis with literature data highlights the favorable performance of IL1, IL2 and IL3 in terms of Henry’s Law constants for CO_2_ absorption. Despite their impressive performance, further investigation into other ionic liquid formulations, such as 1-octyl-3-methyl imidazolium bis(trifluoromethyl) imide, may provide valuable insights into optimizing gas absorption processes.

Overall, the detailed investigation provides valuable insights into the thermodynamics of gas solvation in IL1, IL2 and IL3, offering potential avenues for further research and optimization in gas separation technologies. The observed trends underscore the promise of fluorinated ionic liquids for CO_2_ absorption applications, while also suggesting the need for additional studies to elucidate the underlying molecular mechanisms and optimize the performance of these systems.

## Figures and Tables

**Table 1:** CO_2_ solubility data in IL1, Il2 and IL3, respectively.

IL1					
303.15 K		323.15 K		343.15 K	
$x_{CO_{2}}$	Pressure (MPa)	$x_{CO_{2}}$	Pressure (MPa)	$x_{CO_{2}}$	Pressure (MPa)
0.034	0.0997	0.026	0.1003	0.020	0.0999
0.065	0.1998	0.051	0.2002	0.040	0.1999
0.094	0.2994	0.073	0.2999	0.057	0.2994
0.122	0.4002	0.092	0.4003	0.073	0.3997
					
0.148	0.5004	0.111	0.4989	0.090	0.4999
0.172	0.5988	0.130	0.5990	0.106	0.5994
0.195	0.7003	0.149	0.7006	0.121	0.7011
0.217	0.8003	0.167	0.8002	0.136	0.7994
0.238	0.9000	0.184	0.8995	0.151	0.9007
0.259	0.9999	0.201	0.9998	0.164	1.0004
0.278	1.1000	0.217	1.0990	0.176	1.1005
0.296	1.1990	0.231	1.1990	0.189	1.1998
0.314	1.3000	0.246	1.3010	0.200	1.2992
0.329	1.4000	0.262	1.3990	0.213	1.3989
0.345	1.4995	0.276	1.4991	0.224	1.5003
IL2					
323.15 K		333.15 K		343.15 K	
$x_{CO_{2}}$	Pressure (MPa)	$x_{CO_{2}}$	Pressure (MPa)	$x_{CO_{2}}$	Pressure (MPa)
0.026	0.0998	0.024	0.0999	0.022	0.1003
0.053	0.1999	0.049	0.1997	0.044	0.2009
0.077	0.2999	0.070	0.2985	0.061	0.2993
0.098	0.3999	0.089	0.4001	0.078	0.3996
0.118	0.5005	0.108	0.5008	0.094	0.4988
0.139	0.5991	0.127	0.5987	0.111	0.5990
0.157	0.7006	0.143	0.6992	0.127	0.7008
0.174	0.8010	0.160	0.8009	0.143	0.7998
0.193	0.8993	0.176	0.8997	0.158	0.8997
0.211	1.0006	0.193	0.9996	0.172	1.0007
0.228	1.0998	0.207	1.1006	0.187	1.0997
0.245	1.2002	0.223	1.1998	0.202	1.1997
0.261	1.2999	0.240	1.2995	0.216	1.2991
0.272	1.3999	0.255	1.4165	0.227	1.3989
0.289	1.5071	0.264	1.4991	0.238	1.5003
IL3					
303.15 K		323.15 K		343.15 K	
$x_{CO_{2}}$	Pressure (MPa)	$x_{CO_{2}}$	Pressure (MPa)	$x_{CO_{2}}$	Pressure (MPa)
0.034	0.0999	0.025	0.0999	0.016	0.0986
0.066	0.1998	0.051	0.1998	0.037	0.1997
0.096	0.3004	0.075	0.2996	0.056	0.2999
0.124	0.4004	0.097	0.4003	0.074	0.4000
0.151	0.5009	0.115	0.5001	0.089	0.4941
0.176	0.5994	0.136	0.6005	0.106	0.6003
0.200	0.7012	0.155	0.6987	0.124	0.6991
0.222	0.8006	0.173	0.7992	0.138	0.7956
0.243	0.8988	0.192	0.9007	0.154	0.8949
0.267	0.9937	0.208	0.9996	0.169	1.0003
0.284	1.1005	0.226	1.1007	0.186	1.1251
0.303	1.2000	0.243	1.2004	0.198	1.2005
0.321	1.3002	0.257	1.3005	0.211	1.3053
0.341	1.4084	0.276	1.4219	0.227	1.4093
0.353	1.5013	0.288	1.5003	0.238	1.4990

Standard uncertainty u(x) = 0.006, standard uncertainty u(T) = 0.1 K, standard uncertainty u(P) = 0.0008 MPa.

**Table 2:** Optimized binary interaction parameters for vdW1, vdW2 and WS-NRTL with their corresponding average absolute deviation percentages (AAD %).

Ionic Liquids + CO_2_	Temperature (°C)	Binary Interaction Parameter (k_12_)				% AAD
IL1	30	−0.0711				7.52
	50	−0.0857				6.37
	70	−0.1090				5.68
IL2	50	−0.1030				6.39
	60	−0.1200				6.57
	70	−0.1310				5.67
IL3	30	−0.0737				6.85
	50	−0.0956				5.43
	70	−0.1160				3.14
Ionic Liquids + CO_2_	Temperature (°C)	Binary Interaction Parameter				% AAD
		(k_12_)		(l_12_)		
IL1	30	−0.0029		0.0139		0.38
	50	−0.0081		0.0149		1.01
	70	−0.0107		0.0173		0.63
IL2	50	−0.0372		0.0134		0.96
	60	−0.0455		0.0143		1.04
	70	−0.0493		0.0149		1.49
IL3	30	−0.0166		0.0103		6.85
	50	−0.0347		0.0099		0.87
	70	−0.0717		0.0066		1.48
Ionic Liquids + CO_2_	Temperature (°C)	Binary Interaction Parameter				% AAD
		(k_12_)	(τ_12_)		(τ_21_)	
IL1	30	0.8930	−0.4706		0.0017	0.41
	50	0.8619	−0.6007		0.1619	0.87
	70	0.8039	−0.9079		0.6283	0.59
IL2	50	0.7568	−0.4833		−0.1106	0.97
	60	0.7212	−0.4676		−0.1439	1.00
	70	0.7013	−0.5794		0.0076	1.34
IL3	30	0.9156	0.1346		−0.6906	0.43
	50	0.8161	−0.4606		−0.0019	0.85
	70	0.6969	−0.3518		−0.0001	1.53

$k_{12}$ = $k_{21}$. $l_{12}$ = $l_{21}.$

**Table 3:** Henry’s Law constant, enthalpy and entropy of solvation between CO_2_ and ILs.

Ionic Liquid (ILs)	Henry’s Law Constant (MPa)			$∆H^{\infty }$ (kJ/mol)	$∆S^{\infty }$ (KJ/Kmol·K)
	T = 30 °C	T = 50 °C	T = 70 °C		
IL1	2.79	3.83	4.90	−12.80	−37.78
	T = 50 °C	T = 60 °C	T = 70 °C		
IL2	3.66	4.05	4.58	−10.33	−31.04
IL3	T = 30 °C	T = 50 °C	T = 70 °C		
	2.71	3.82	5.13	−13.79	−42.80

**Table 4:** Detailed list of all ionic liquids.

Ionic Liquid	Nomenclature	Acronym	Structure
1-Decyl-3-MethylimidazoliumBis (Trifluromethylsulfonyl Imide) (≥98.0%, water: 8 ppm) * CAS Number: 433337-23-6	C_15_H_26_F_6_N_3_O_4_S_2_	IL1	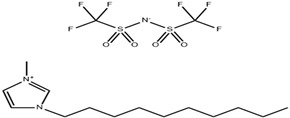
1-Hexadecyl-3-Methyl imidazoliumBis (Trifluromethylsulfonyl Imide) (≥98.0%, water: 146 ppm) * CAS Number: 404001-50-9	C_21_H_40_F_6_N_3_O_4_S_2_	IL2	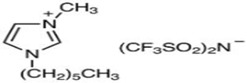
Triethyl tetradecyl Ammonium Bis Bis (Trifluromethylsulfonyl Imide) (≥98.0%, water: 102 ppm) * CAS Number: n/a	C_20_H_44_F_6_N_2_O_4_S_2_	IL3	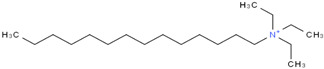
			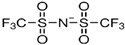

* Provided by manufacturer (IoLiTec, Heilbronn, Germany).

**Table 5:** Density of the ILs used in this work.

Temperature (K)	Density (g/cm^3^)		
	IL1	IL2	IL3
303.15	1.2727	-	1.1608
308.15	1.2684	-	1.1569
313.15	1.2640	-	1.1529
318.15	1.2597	-	1.1488
323.15	1.2553	1.1693	1.1446
328.15	1.2510	1.1652	1.1405
333.15	1.2465	1.1612	1.1362
338.15	1.2418	1.1572	1.1317
343.15	1.2368	1.1532	1.1270

Standard uncertainty u (ρ) = 0.0002 g/cm^3^; standard uncertainty u (T) = 0.01 K; standard uncertainty u (P) = 0.8 kPa.

**Table 6:** Critical properties.

Component	Tc (K)	Pc (Bar)	ω
IL1	1345.1	18.700	0.5741
IL2	1195.4	18.347	0.9176
IL3	1207.7	14.265	1.1367
CO_2_	304.1	73.800	0.2390

## Data Availability

All measured experimental data are reported in the manuscript.
